# Items

**Published:** 1875-01

**Authors:** 


Notice of Examination. Candidates for the position of resident physician at the Buffalo General Hospital, are requested to send their names to the Secretary of the Staff, Dr. C. Diehl, No. 32 West Genesee street, on or before the 22d of February, 1875, when they will be informed of the time and place of the examination, S. F. Mixer, President of Staff.——Crowded Out. Owing to the press of other matter, the transactions of the Buffalo Medical Association were crowded out of this issue, several other interesting papers were also awaiting publication, we ask our contributors to be a little patient, and assure them that their articles will appear in due time.——The Medical Record. This valuable Journal is now published weekly, beginning with January 1st.——New Enterprise. Messrs. G. P. Putnam's Sons announce that they will shortly commence the publication of a series of American Clinical Lectures, under the editorial supervision of Dr. E. C. Seguin, of New York. These lectures are to be sold singly for the first year at prices varying from thirty to fifty cents. The first one will be ready about Feb. 1st, and is to be by Dr. L. A. Sayre on Disease of the Hip Joint.——A Generous Offer. The Editor of the *Richmond and Louisville Medical Journal*, and the *American Medical Weekly* offers to each subscriber of the former journal twelve engraved portraits of distinguished European and American physicians, and to subscribers of the "Weekly" a portrait in each of the two volumes published during the year; the portraits in the "monthly" and "weekly" are different.——Pepsine. In the use of pepsine it is essential in order to obtain any results that the article should be pure, and carefully prepared, these indications are fully met in an article prepared by Mr. Thompson of this city. His pepsine has been found to meet all the usual tests applied to it, and in cases where it has been employed has obtained the approval of those using it——Resigned. Dr. J. Marion Sims has resigned his position as one of the Surgeons to the Womans Hospital, New York; Dr. Fordyce Barker has been appointed to fill the vacancy thus created.——Vivisection. Every member of the profession should read Prof. J. C. Dalton's recent book, Experimentation on Animals as a means of knowledge of Physiology, Pathology and Practical Medicine.

